# Maternal Docosahexaenoic Acid Supplementation Alters Maternal and Fetal Docosahexaenoic Acid Status and Placenta Phospholipids in Pregnancies Complicated by High Body Mass Index

**DOI:** 10.3390/nu16172934

**Published:** 2024-09-02

**Authors:** Katie L. Bidne, Karin Zemski Berry, Mairead Dillon, Thomas Jansson, Theresa L. Powell

**Affiliations:** 1 Departments of Obstetrics and Gynecology, University of Colorado Anschutz Medical Center, Aurora, CO 80045, USAthomas.jansson@cuanschutz.edu (T.J.); 2 Departments of Medicine, University of Colorado Anschutz Medical Center, Aurora, CO 80045, USA; 3 Departments of Pediatrics, University of Colorado Anschutz Medical Center, Aurora, CO 80045, USA

**Keywords:** fetal development, maternal–fetal exchange, choline, fatty acids, omega-3

## Abstract

Introduction: An optimal fetal supply of docosahexaenoic acid (DHA) is critical for normal brain development. The relationship between maternal DHA intake and DHA delivery to the fetus is complex and is dependent on placental handling of DHA. Little data exist on placental DHA levels in pregnancies supplemented with the recommended dose of 200 mg/d. Our objective was to determine how prenatal DHA at the recommended 200 mg/d impacts maternal, placental, and fetal DHA status in both normal-weight and high-BMI women compared to women taking no supplements. Methods: Maternal blood, placenta, and cord blood were collected from 30 healthy pregnant women (BMI 18.9–43.26 kg/m^2^) giving birth at term. Red blood cells (RBCs) and villous tissue were isolated, and lipids were extracted to determine DHA content by LC-MS/MS. Data were analyzed by supplement group (0 vs. 200 mg/d) and maternal BMI (normal weight or high BMI) using two-way ANOVA. We measured maternal choline levels in maternal and cord plasma samples. Results: Supplementation with 200 mg/d DHA significantly increased (*p* < 0.05) maternal and cord RBC DHA content only in pregnancies complicated by high BMI. We did not find any impact of choline levels on maternal or cord RBC phospholipids. There were no significant differences in total placental DHA content by supplementation or maternal BMI (*p* > 0.05). Placental levels of phosphatidylinositol (PI) and phosphatidic acid containing DHA species were higher (*p* < 0.05) in high-BMI women without DHA supplementation compared to both normal-BMI and high-BMI women taking DHA supplements. Conclusion: Maternal DHA supplementation at recommended doses cord increased RBC DHA content only in pregnancies complicated by higher BMI. Surprisingly, we found that obesity was related to an increase in placental PI and phosphatidic acid species, which was ameliorated by DHA supplementation. Phosphatidic acid activates placental mTOR, which regulates amino acid transport and may explain previous findings of the impact of DHA on placental function. Current recommendations for DHA supplementation may not be achieving the goal of improving fetal DHA levels in normal-weight women.

## 1. Introduction

Docosahexaenoic acid (22:6n-3; DHA), an omega-3 fatty acid, is of particular interest in pregnancy due to its positive associations with pregnancy outcomes [1], fetal brain development [2], and offspring cognitive function [3]. The maternal liver can synthesize DHA from alpha linoleic acid via desaturases and elongases. However, both the placenta and the fetus lack this capability. Therefore, fetal DHA status is dependent on both maternal DHA levels and placental metabolism and transfer of this long chain polyunsaturated fatty acid (LCPUFA). While essential fatty acids (n-3 a-linolenic acid and n-6 linoleic acid) are thought to be converted to their long-chain polyunsaturated forms (n-3 DHA and n-6 Arachidonic acid), studies using stable isotopes have indicated that the conversion of a-linolenic acid to DHA is highly inefficient [4]. This suggests that the dietary intake of pre-formed DHA is critical at all stages of life but, in particular, during pregnancy when maternal dietary supplies and placental transfer are critical for fetal development. Due to the importance of pre-formed DHA during pregnancy, various agencies have recommended consumption of 200–300 mg DHA daily either from food sources such as fish or by supplementation during pregnancy [5] to ensure adequate supply to the developing fetus. Data analyzed from NHANES have found that pregnant women in the US consume approximately 67 mg DHA daily [6], significantly lower than the recommended intake making supplementation critical.

Research into DHA supplementation in pregnancy has focused on offspring neurodevelopment and prevention of preterm birth. DHA is important for proper myelination [7] and has been linked to functions in the brain, including modulating membrane fluidity, prevention of apoptosis, and regulating nociception [8]. However, studies on the impact of DHA supplementation during pregnancy on offspring brain function have produced mixed results, with some studies demonstrating a benefit and others observing no effects [9,10]. Importantly, supplementation and assessments of neurological function varied in each study, making generalizations difficult. Supplementation with 600–800 mg/d of DHA has been reported to decrease rates of preterm birth [1,9]. Previous studies point to a complex regulation of DHA status in maternal, placental, and fetal compartments. In a study comparing red blood cell (RBC) DHA status in maternal-newborn dyads, it was found that a dose of 800 mg/d was inadequate to achieve DHA sufficiency in some newborns [11]. A complication of previous assessments of DHA in pregnancy is using a percentage of total fatty acids as the unit for DHA content. This measure only provides information on relative changes in fatty acid composition but not changes in the concentration of DHA. The impact of recommended doses of DHA supplementation in pregnancy on placental and fetal DHA levels is not well established. Moreover, obesity has been reported to be associated with reduced maternal DHA status [12,13], which may suggest a greater need for supplementation in these pregnancies. 

The availability of choline is believed to influence maternal and fetal DHA status, especially in membrane phospholipid levels such as RBC. In pregnant women taking 200 mg/d DHA with or without 500 mg/d choline supplement, the women who also consume supplemental choline had elevated RBC DHA status. However, there were no differences in placental or fetal RBC DHA status [14]. The role of maternal choline in phospholipid DHA incorporation in maternal, placental, and fetal compartments has not been adequately studied. 

The importance of DHA in fetal development is underscored by the preferential transport of this fatty acid by the placenta when compared to other fatty acids [15]. While the mechanisms for transplacental DHA transport are not fully understood, it is currently believed that the placenta can transport DHA in two forms: as a non-esterified fatty acid or a lysophospholipid [16]. To generate the lysophospholipid form (a single acyl chain) requires that DHA is incorporated into phospholipids in the syncytiotrophoblast and then converted into the lysophospholipid form by phospholipases. We have previously reported expression of a lysophospholipid transporter, Major Facilitator Superfamily Domain containing 2a (MFSD2a), in human placenta, suggesting lysophospholipid DHA is transported to the fetus [16]. We have also demonstrated in a mouse model of pregnancy that a specific reduction in placental MFSD2a expression, with no changes in the fetal brain expression of the transporter, caused a reduction in fetal brain size and phospholipid DHA content [17].

There have been a few reports comparing obese women with and without DHA supplementation. A study comparing a high DHA dose (2 g/d DHA plus EPA) to placebo in obese and overweight pregnant women demonstrated that DHA supplementation decreases total placenta lipid per gram tissue and reduces expression of enzymes that are responsible for triglyceride synthesis [12]. Our group has previously shown that increased placental DHA content in pregnancies complicated by obesity was associated with decreased abundance and activity of amino acid transporters, increased fatty acid and glucose transporters, reduced inflammatory markers, and insulin signaling [18]. Additionally, we have demonstrated that DHA inhibits mTOR activity in cultured trophoblast cells [19]. When these changes are considered alongside obesity-induced metabolic alterations in pregnancy, such as maternal hyperinsulinemia [20] and placental lipotoxicity [21], the necessity to understand how DHA supplementation may interplay with obesity becomes apparent. 

In this study, we sought to investigate how standard DHA-containing prenatal supplements impact maternal, placental, and fetal DHA status in normal-weight and high-BMI pregnant women. We hypothesized that women consuming a prenatal supplement containing 200 mg of DHA have elevated maternal, placental, and fetal DHA status and that these increases are greater in high-BMI compared to normal-weight women. 

## 2. Methods

### 2.1. Sample Collection

This was an observational study, not a randomized intervention. We recruited 30 pregnant women at the University of Colorado Hospital Labor and Delivery unit with informed consent (IRB number 14-1073). Subjects with pre- or early-pregnancy BMI of 18.5–45 kg/m^2^, term delivery, singleton pregnancy, fetal growth appropriate for gestational age, and no maternal anemia or other pregnancy complications were included in the study. Our objective was to determine the impact of spontaneous DHA supplementation in pregnancy on maternal, fetal, and cord DHA status and to determine whether maternal choline levels and obesity impacted DHA status at the end of pregnancy. All women were consented after admission to the hospital for delivery by our trained labor and delivery research staff. They were given time to read the consent and consult with family or medical staff before signing the consent. Maternal blood was taken prior to delivery, and cord blood and placenta samples were collected immediately after delivery. Data retrieval from their electronic medical records was also performed by our trained research staff and only deidentified data were entered into a password-protected secure REDCap database for access by the laboratory researchers. Likewise, only deidentified samples were collected by laboratory staff for use in all analyses. Therefore, only hospital research staff had access to identifying information to protect the identity and privacy of all subjects. 

Maternal blood, cord blood, and placenta were collected and processed at the time of delivery. Prenatal vitamin and DHA supplementation were recorded at the time of consent. Blood samples were collected in EDTA tubes and centrifuged at 1500 rcf for 15 min at 4 °C to separate plasma and red blood cells (RBCs). Plasma was aliquoted and flash-frozen in liquid nitrogen. Approximately 1 mL of RBC pellets were added to a new tube with sterile saline and centrifuged again. Supernatant was removed and the process repeated. One 1mL sterile saline was added to the cleaned pellet, followed by vortexing, aliquoting, and flash freezing in liquid nitrogen. Samples were stored at −80 °C until the time of analysis. Placental villous biopsies were dissected, rinsed in sterile saline, flash-frozen in liquid nitrogen, and stored at −80 °C until the time of analysis. 

### 2.2. Total DHA Quantification

The protein content of maternal and cord RBCs was determined by Nanodrop, and a volume equivalent to 1 mg protein was utilized for lipid extractions. Placental tissue was homogenized in water using a bead beater and protein quantified via BCA (Thermo Fisher, Waltham, MA, USA). Homogenates equivalent to 5 mg tissue was utilized for lipid extractions. Samples were subjected to a modified Bligh and Dyer lipid extraction. Briefly, the samples were added to acidified methanol, spiked with 1 μg PC 19:0/19:0 to calculate extraction efficiency, and had chloroform and water added. Centrifugation was performed to separate the phases, and the chloroform layer was removed. To this, 50 ng d_5_22:6 and 100 ng d_8_18:0 were added as an internal standard for quantification. The samples were dried down and resuspended in ethanol. Sodium hydroxide was added to samples that were subsequently heated at 95 °C for 1 h to saponify lipids. Samples were then acidified using hydrochloric acid, extracted with iso-octane, dried down, and derivatized using 1% pentafluorobenzyl bromide and 1% *N*,*N*-diisopropylethylamine, both in acetonitrile. After incubation, samples were dried down, resuspended in iso-octane, aliquoted into vials, and subjected to mass spectrometry analysis. Quantification of DHA was conducted under negative ion chemical ionization (NICI) mode using an Agilent 7890B gas chromatograph coupled to an Agilent 5977B single quadrupole mass spectrometer (Agilent Technologies, Santa Clara, CA, USA). Injections were executed with an Agilent 7693A Autoinjector into a split–splitless liner setup to a non-polar capillary GC column (HP-5, 5% Phenyl-methylpolysiloxane). Chromatographic analysis was completed using Agilent’s MassHunter Qualitative Analysis (Version B.08.00) integration, and quantification was performed with Quantitative Analysis (Version B.09.00) software. An internal standard mixture was added to each sample to determine the absolute concentration using calibration curves containing DHA, and fatty acid 19:0 from 2000 ng to 0.32 ng with 50 ng d_5_22:6 and 100 ng d_8_18:0 was run alongside samples to determine the extraction efficiency and concentration of DHA in each sample.

### 2.3. Phospholipid Analysis

Placenta samples (~30 mg) were homogenized in water and subjected to lipid extraction and liquid chromatography tandem mass spectrometry (LC-MS/MS) at the University of Colorado NORC Lipidomics Core facility. Phosphatidyl choline (PC), ethanolamine (PE), inositol (PI), and phosphatidic acid species were quantified. For each sample, the ~15 mg of tissue was brought up to a volume of 750 uL with water, and 900 uL methanol containing 0.01% butylated hydroxytoluene was added. An internal standard cocktail containing 17:0_17:0 PE (1000 pmol), 17:1 LPE (100 pmol), 19:0_19:0 PC (2000 pmol), d_7_-18:1 LPC (200 pmol), 17:0_17:0 PG (250 pmol), 17:1 LPG (25 pmol), 17:0_17:0 PS (300 pmol), 17:1 LPS (100 pmol), 17:0_17:0 PA (20 pmol), and 17:1 LPA (20 pmol) was added, and lipid extraction was performed by the addition of methyl-*tert*-butyl ether (3 mL) as previously described [22]. Samples were injected onto an LC-MS/MS system (Sciex 3200 triple quadrupole mass spectrometer), and mass spectrometric analysis was performed in the negative ion mode using multiple reaction monitoring (MRM). Quantitative results were determined using stable isotope dilution with standard curves for saturated and unsaturated phospholipid compounds as previously described. The precursor ions monitored were the molecular ions [M-H]^−^ for PE, LPE, PS, LPS, PG, LPG, PA, LPA, PI, and LPI, and the acetate adducts [M+CH_3_COO]^−^ for PC and LPC. The product ions analyzed after collision-induced decomposition were the carboxylate anions corresponding to the acyl chains. 

### 2.4. Choline Analysis

Plasma choline concentrations in maternal and cord plasma were determined by ultra-high performance liquid chromatography (UHPLC) coupled with high-resolution mass spectrometry (MS) at the University of Colorado Metabolomics Core, as previously described [23]. 

### 2.5. Statistics

The data were analyzed by maternal BMI and DHA supplementation status. Maternal BMI of 18.5–24.9 kg/m^2^ was considered control, and BMI of >25 was considered high BMI. Supplementation is denoted as 0 or 200, representing the DHA dose in mg/d in the reported prenatal supplement. This resulted in four groups: control 0 mg/d (n = 10), control 200 mg/d (n = 7), high BMI 0 mg/d (n = 6), and high BMI 200 mg/d (n = 10). 

Maternal demographics, maternal RBC DHA, cord RBC DHA, placenta DHA, and placenta lipidomic data were analyzed using GraphPad Prism via two-way ANOVA with Tukey’s adjustment for multiple comparisons. ANOVA models were created to predict maternal RBC DHA, cord RBC DHA, PC, and PE using choline, BMI category, and the presence of supplementation. All models were initially run with the interaction of BMI category and supplementation as a predictor. If the interaction was not significant, the model was re-run without the interaction in order to interpret the main effects. *p*-values < 0.05 were considered statistically significant.

## 3. Results

### 3.1. Clinical Characteristics

Selected characteristics of the study population are shown in Table 1. The groups consuming 200 mg/d DHA had a significantly higher maternal age compared to those without supplementation. By study design, maternal BMI was significantly different between the control and high-BMI groups. All other variables were not different between groups. 

### 3.2. Maternal, Fetal, Placental DHA Status

**Figure 1:**
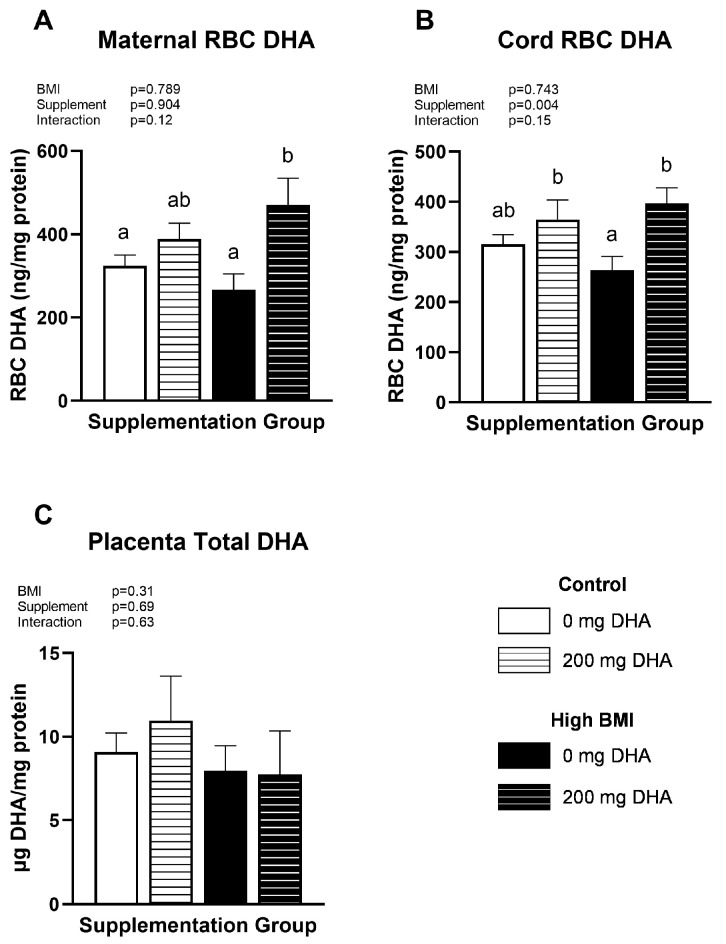
(**A**) Maternal RBC, (**B**) Cord RBC, and (**C**) Placental DHA status. Different letters indicate statistical differences between groups via two-way ANOVA with multiple comparisons and Tukey’s modification, *p* < 0.05.

Maternal and fetal DHA status were assessed in red blood cells (RBCs) from maternal venous blood and umbilical cord blood, respectively. Interestingly, DHA concentrations in RBC from normal-BMI women consuming 200 mg/d DHA and those without supplementation were not statistically different (Figure 1A). We did, however, find a significant elevation in RBC DHA in high-BMI subjects consuming 200 mg/d compared to those with no supplementation (*p* < 0.05, Figure 1A). Similarly, when comparing cord RBC DHA, we found no difference in normal-weight groups as a result of supplementation but did find a significant elevation in cord blood RBC of high-BMI subjects who were consuming 200 mg/d compared to obese subjects without supplementation (*p* < 0.05, Figure 1B). In contrast, total DHA concentrations in placental tissue were not influenced by maternal BMI or supplementation status (Figure 1C). 

### 3.3. Placental DHA-Phospholipid Concentrations

**Figure 2:**
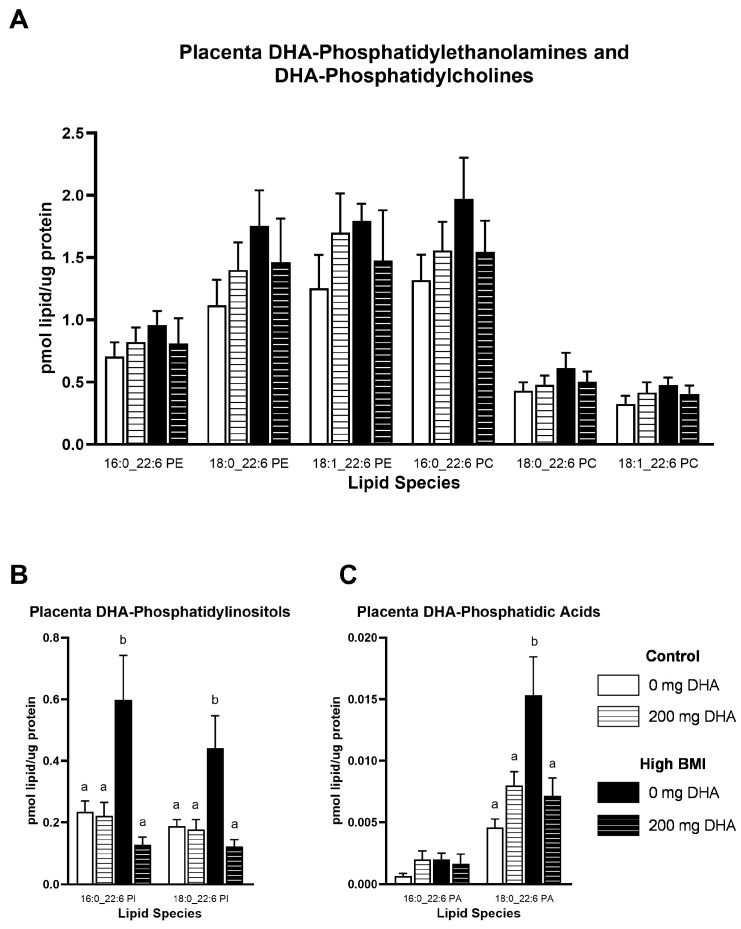
Placenta DHA-containing (**A**) phosphatidylethanolamines and phosphatidylcholines, (**B**) phosphatidylinositols, and (**C**) phosphatidic acids. Different letters indicate statistical differences between groups via two-way ANOVA with multiple comparisons and Tukey’s modification, *p* < 0.05.

Because phospholipids may play a role in the ability of the placenta to transport DHA to the fetus, we quantified phospholipids containing DHA in the placenta. We did not observe any differences due to maternal BMI or supplementation in any of the DHA-containing phosphatidylethanolamine (PE) or phosphatidylcholine (PC; Figure 2A) lipids in placental tissue. When we quantified placenta DHA-containing phosphatidylinositol (PI), we found a significant interaction of maternal BMI and supplementation in two species of PI, 16:0_22:6 and 18:0_22:6, driven by a significant increase in these phospholipids in the high-BMI group without supplementation compared to all other groups (Figure 2B, *p* < 0.05). We further determined the concentrations of placental phosphatidic acid, a key precursor to phospholipids, and found a significant interaction of BMI and supplementation with 18:0_22:6 PA but not 16:0_22:6 PA. This change was also driven by an increase in PA containing DHA in the high-BMI with no supplement group compared to all other groups (Figure 2C, *p* < 0.05). 

### 3.4. Placental Phospholipids

**Figure 3:**
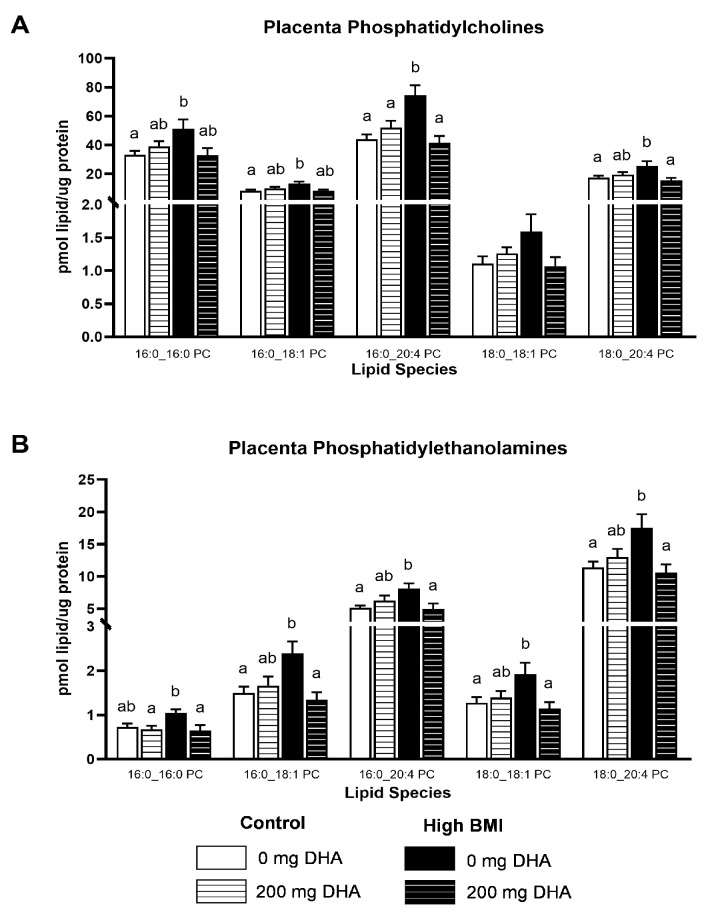
Selected placenta: (**A**) phosphatidylcholines and (**B**) phosphatidylethanolamines. Different letters indicate statistical differences between groups via two-way ANOVA with multiple comparisons and Tukey’s modification, *p* < 0.05.

**Figure 4:**
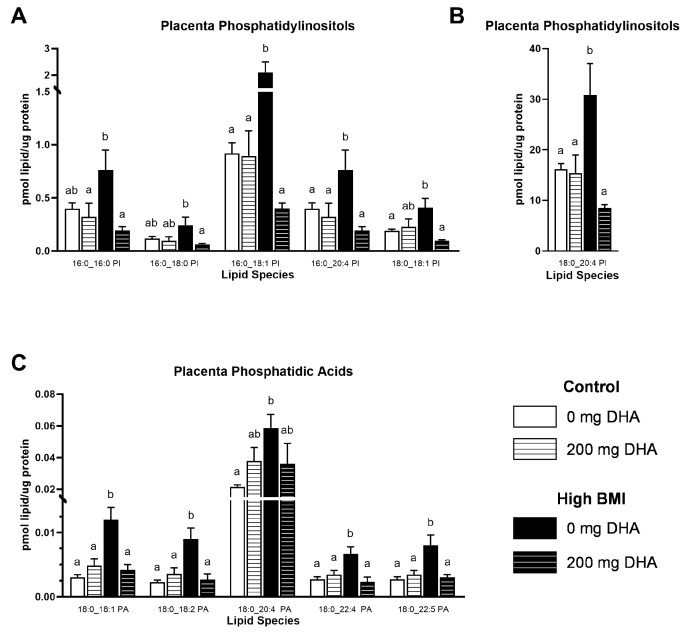
Selected placenta: (**A**,**B**) phosphatidylinositols and (**C**) phosphatidic acids. Different letters indicate statistical differences between groups via two-way ANOVA with multiple comparisons and Tukey’s modification, *p* < 0.05.

Given the striking increases in the abundance of specific phospholipids in the high-BMI non-supplemented group compared to others, we elected to broaden our analysis to other phospholipids in the placenta. In numerous PC and PE species (Figure 3A,B, Supplemental Tables S1 and S2), we observed that placentas from high-BMI non-supplemented pregnancies had elevated phospholipid content compared to placentas from the supplemented group and both normal-BMI groups (*p* < 0.05). There were no differences due to DHA supplementation in the normal-BMI groups. Similarly, in PI phospholipids (Figure 4A,B, Supplemental Table S3), we observed a similar pattern of increased concentrations of several PI species in placentas from high-BMI non-supplemented women compared to all other groups (*p* < 0.05). Interestingly, in the placental phosphatidic acid species (Figure 4C, Supplemental Table S4), we found the same pattern of increase in the high-BMI non-supplemented group compared to all the others (*p* < 0.05), but this was limited to the 18:0-containing species. 

We also determined correlations of the individual phosphatidic acid species with their respective PC, PE, and PI counterparts (Table 2) Given that phosphatidic acid is a precursor to each of these phospholipids through the de novo synthesis pathway, we wanted to investigate how the abundance of this precursor molecule impacted abundance of the downstream phospholipids. We found that the phosphatidic acid species (Supplemental Table S4) correlated to their PE, PC, and PI counterparts but only in the 18:0-containing species. 

### 3.5. Choline Status

**Figure 5:**
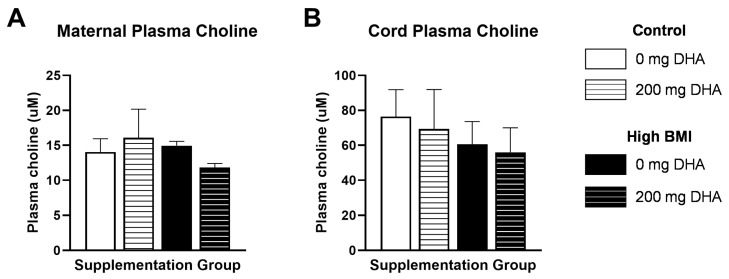
**(A)** Maternal and (**B**) cord plasma choline concentrations. No differences between any groups via two-way ANOVA with Tukey’s adjustment.

**Figure 6:**
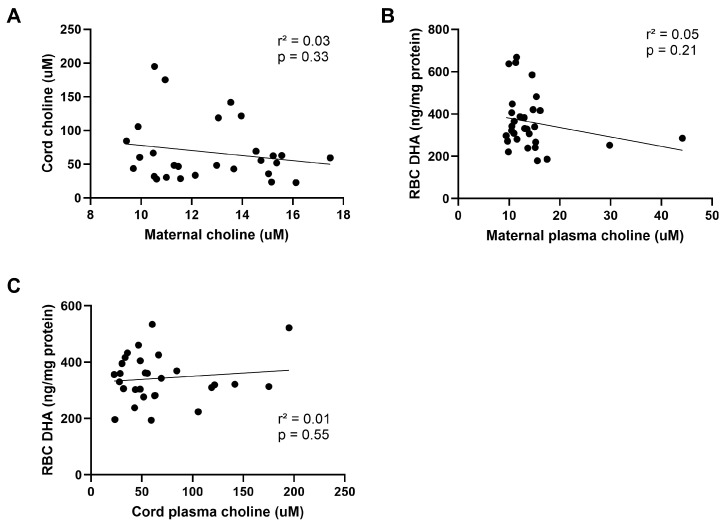
Correlations of (**A**) maternal and cord plasma choline, (**B**) Maternal RBC DHA and maternal plasma choline, and (**C**) Cord RBC DHA and cord plasma choline. Data were analyzed using Pearson’s correlation.

Other studies have demonstrated a relationship between maternal DHA status and circulating choline levels. Therefore, we quantified plasma choline in both maternal and cord plasma. We did not find any significant differences between groups (Figure 5A,B) in either maternal or cord plasma choline. We analyzed whether maternal choline was associated with RBC DHA levels in normal- and high-BMI women. The interaction of BMI category and supplementation was not significant, so the model was re-run without the interaction. Supplementation was the only statistically significant predictor of maternal RBC DHA (*p* = 0.006). A similar model was applied using cord plasma choline, BMI category, and supplementation to predict cord RBC DHA. Supplementation was the only statistically significant predictor of cord RBC DHA (*p* = 0.002). Further, we did not observe significant correlations between maternal and cord plasma choline (Figure 6A), maternal RBC DHA and maternal plasma choline (Figure 6B), or cord RBC DHA and cord plasma choline (Figure 6C).

## 4. Discussion

In this study, we examined the effect of 200 mg/d prenatal DHA supplementation in women who were normal-weight or high-BMI on maternal, fetal, and placental DHA status. To our knowledge, this is the first study to demonstrate the impact of standard DHA supplementation in pregnancy on placental DHA status and allows for comparison between normal- and high-BMI women. Our hypothesis was that women consuming a prenatal supplement containing 200 mg of DHA have elevated maternal, placental, and fetal DHA status and that these increases are greater in high-BMI compared to normal-weight women. 

Surprisingly, we found that supplementation with the recommended 200 mg/d DHA did not result in a significant increase in either maternal or cord RBC DHA concentrations in the normal-BMI group. However, high-BMI women consuming 200 mg/d had elevated DHA in both maternal and cord RBC compared to the women without supplementation. Though not statistically different than the respective normal-weight groups, in both maternal and cord samples the high-BMI non-supplemented and supplemented groups were numerically the lowest and highest averages, respectively. One factor that could contribute to this would be the DHA status when entering pregnancy, and the duration and compliance with supplementation during pregnancy. These variables were not assessed in this study. Others have reported that obesity is associated with decreased RBC DHA status compared to a normal weight even when DHA consumption was equal [24]. The potential for a lower DHA status when entering pregnancy due to obesity could explain why supplementation of the recommended DHA dose of 200 mg/d appeared to be more effective in the high-BMI subjects in this study. This would suggest that consuming the recommended 200 mg/d DHA may be insufficient to increase DHA status in normal-weight women. However, our limited sample size and lack of information on duration and compliance with supplementation make it clear that carefully controlled trials with a larger sample size and longitudinal sampling are needed to confirm this finding. 

Maternal choline availability has been suggested to be important in determining pregnancy DHA status [14]. This may be particularly true in light of recent evidence demonstrating that DHA in lysophosphatidylcholine is likely transported to the fetus through MFSD2a transporter localized to the basal or fetal facing plasma membrane of the syncytiotrophoblast. The transport of DHA in this form would require choline as a head group for the incorporation of DHA into placental phosphatidylcholine followed by phospholipase activity to generate lysophosphatidylcholine containing DHA. We examined the levels of choline in our four groups, both in maternal and cord blood, and found no significant difference. In the modeling of three variables, maternal BMI, choline, and DHA supplementation status, only DHA supplementation predicted DHA levels in RBC samples.

We found no significant differences in total placental DHA concentrations between the four groups. Phospholipid species containing DHA were impacted only in the phosphatidylinositol class where DHA supplementation resulted in lower 16:0_22:6 PI and 18:0_22:6 PI in pregnancies of high-BMI mothers. This was surprising as we expected supplementation to increase phospholipid DHA in the placenta. Interestingly, we consistently found the impact of obesity on placental phospholipids with many non-DHA-containing phospholipids to be highest in placentas from the high-BMI non-supplemented group. DHA supplementation with 200 mg/d ameliorated the increased phospholipid content in placentas of these mothers. In fact, of the quantified placental phospholipids, we observed this same pattern of elevated phospholipids in high-BMI pregnancies and reduction with DHA supplementation in 10/25 PE species, 12/23 PC species, and 18/20 PI phospholipid species. Though we observed a consistent pattern of higher placental concentrations of many phospholipids in the high-BMI non-supplemented group, the PI species were impacted to the greatest degree. Placental phosphatidyl inositol concentrations in high-BMI women without DHA supplementation ranged from 42 to 90% higher than all other groups studied, whereas in the PE and PC phospholipids, this difference was 16–51% and 17–47%, respectively, higher in placentas of women with elevated BMI. 

**Figure 7:**
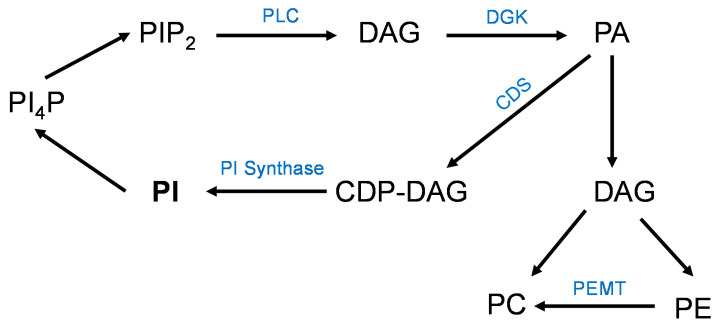
Phosphatidylinositol pathway showing PI is sequentially metabolized into phosphoinositol-4-phosphate (PI4P) and phosphatidylinositol 4,5-bisphosphate (PIP2). PIP2 can be converted by phospholipase C (PLC) into diacylglycerol (DAG), which is acted upon by diacyglycerol kinase (DGK) to form phosphatidic acid (PA), which can be used for the generation of PI, PC, and PE phospholipids.

Phospholipids have somewhat overlapping intermediates in their synthesis/metabolism and we chose to quantify a central one, phosphatidic acid, to understand more about the interplay of these lipid species within the placenta. We observed the same pattern of high phosphatidic acid levels in placental tissue of non-supplemented high-BMI pregnancies when compared to all the other groups, but this was only found in the 18:0-containing species. Further, when we correlated the individual phosphatidic acid species with their corresponding PC, PE, and PI, all the 18:0-containing phosphatidic acid species were significantly correlated with their phospholipid counterparts. We propose that these correlations of the mature phospholipids with their intermediate phosphatidic acid form are related to the metabolism of the phospholipids, specifically the phosphatidylinositol cycle. In this cycle (Figure 7), PI are metabolized into phosphoinositol-4-phosphate (PI_4_P) and phosphatidylinositol 4,5-bisphosphate (PIP_2_). PIP_2_ can be converted by phospholipase C (PLC) into diacylglycerol (DAG), which is converted to phosphatidic acid by diacyglycerol kinase (DGK) [25]. This cycle primarily occurs in the plasma membrane [26]. The DGK enzyme has a preference for 18:0- and 20:4-containing PA species [27]. Therefore, it is not surprising that we found differences in placental 18:0-containing phosphatidic acid in this study. Phosphatidic acid can be acted upon either by CDP-DAG synthases to form CDP-DAG and then PI or by a phosphatidic acid phosphatase for PC and PE synthesis [28]. The PC and PE synthesis primarily occurs in the endoplasmic reticulum and nuclear membranes [29]. We speculate that the mechanism for the increases in placental phospholipids in pregnancies complicated by obesity is the activation of the described PI cycle due to inflammation [30,31,32]. As is clear in Figure 7, Diacyl Glycerol (DAG) is a critical intermediate form in the synthesis of all phospholipids as well as triacylglycerols. This intermediate is also a component of cell membranes and involved in many regulatory pathways, and cellular DAG levels are carefully regulated in multiple cellular compartments [33]. Additional information is needed on the synthesis and regulation of DAG in human placental trophoblast cells.

Obesity is associated with increases in circulating saturated fatty acids [34] and placental inflammation [35]. Saturated fatty acids bind and activate toll-like receptor 4 (TLR4) [36,37]. For downstream inflammatory signaling, the protein MyD88 requires a physical association with PIP_2_ [38], and the activation of TLR4 also activates PLC [39], converting PIP_2_ to DAG. We found that TLR4 knockdown in trophoblast cells blocked the effect of oleic acid on the stimulation of mTOR [37]. Additionally, we found that blocking the synthesis of phosphatidic acid by knocking down acyl transferase enzymes also prevented the activation of mTOR by oleic acid [40]. DHA can inhibit TLR4 signaling [41,42]; therefore, DHA supplementation in obese women may decrease placental TLR4 signaling, reduce placental inflammation, and result in lower PC, PE, PI, and PA species through a reduction of the PI cycle described above. 

Phosphatidic acid has been shown to activate mTOR signaling [43]. In the placenta, increased mTOR signaling has been linked to increased fetal growth rates via the up-regulation of nutrient transporters. Specifically, mTOR activation drives placental amino acid transfer via the up-regulation of System A and L transporters. The activity of these two transport systems was negatively correlated with placental DHA content in our earlier study of obese women supplemented with 800 mg/d DHA [18], perhaps due to a smaller amount of phosphatidic acid in these placentas. Markers of placental inflammation were also reduced with this level of supplementation. In the current study, we found increased placental phosphatidic acid in the non-supplemented high-BMI group, which could be contributing to placental mTOR activation and increased System A and L activity. While there were no differences in birthweight between groups in this study, this was by design, as only pregnancies with fetal weights classified as appropriate for gestational age were included. While outside the scope of this study, additional research is warranted to further understand how increased DHA intake during pregnancy may impact placental function, fetal growth, and birthweight, especially in women who enter pregnancy with elevated BMI.

## 5. Limitations

The limitations of this study include its small sample sizes and a lack of records of DHA supplement usage over gestation as well as dietary recalls to establish dietary DHA consumption in all groups. The examination of the RBC data suggests that pregnancies complicated by high BMI have a non-significant reduction in DHA concentrations in the maternal and fetal compartments. Dietary consumption in our non-supplemented groups is likely similar, and the DHA content of RBCs was not significantly different. Our supplemented subjects were taking their supplements spontaneously, not in relation to enrollment in a study, and, therefore, this may represent population norms for supplement usage, but duration and compliance with supplement usage is an unknown factor in this study. 

## 6. Conclusions

Our data from women who were spontaneously taking DHA supplementation during pregnancy, using perinatal commercially available products containing the recommended DHA level of 200 mg/d, suggests that this dose may not be sufficient to elevate maternal, placental, or fetal DHA concentrations above non-supplemented levels in normal-weight women. However, in high-BMI women, we found that 200 mg/d was sufficient to increase DHA status in the maternal and fetal RBCs. In none of our participants did supplementation increase total DHA content of the placenta. We also demonstrated an obesity-related increased concentration in numerous placental phospholipid species that was ameliorated by DHA supplementation. This increase in placenta phospholipids may be related to low-grade placental inflammation in pregnancies complicated by obesity and could contribute to accelerated fetal growth due to the activation of the mTOR signaling pathway by phospholipids. We have previously shown that DHA inhibits mTOR activity in trophoblast cells, which may indicate that DHA supplementation is a mechanism for reducing mTOR activity in the placentas of pregnancies complicated by obesity by lowering activating phospholipids. We suggest that a detailed analysis of the impact of the recommended dose of 200 mg/d DHA in pregnancy in maternal, fetal, and placental tissues is warranted as this dose did not significantly increase cord RBC or placental DHA content in normal-weight women. 

## Figures and Tables

**Table 1:** Selected maternal demographics. Data shown are mean ± SEM.

	Control		High BMI		*p* Values		
DHA Supplementation (mg/d)	0	200	0	200	BMI	Supp	B*S
N	10	7	6	7			
Maternal Age	30.9 ± 1.97	34.0 ± 2.12	30.0 ± 2.19	35.5 ± 1.23	0.88	0.04	0.56
BMI	22.24 ± 0.57	22.1 ± 0.61	31.93 ± 2.33	30.1 ± 0.92	<0.0001	0.38	0.45
Fetal Sex (M/F)	5/5	2/6	2/4	5/1			

Body Mass Index (BMI), DHA supplementation (Supp), B*S is the interaction term for Body mass index and DHA supplementation by two-way ANOVA.

**Table 2:** Correlations between Phosphatidic acid and corresponding phospholipid species in human placenta tissue.

Correlation with Corresponding Phosphatidic Acid Species		
Species	R^2^	*p* Value
18:0_18:1 PC	0.39	0.0003
18:0_18:2 PC	0.67	<0.0001
18:0_20:4 PC	0.26	0.004
18:0_22:4 PC	0.14	0.04
18:0_22:6 PC	0.32	0.0013
18:0_18:1 PE	0.38	0.0004
18:0_18:2 PE	0.53	<0.0001
18:0_20:4 PE	0.15	0.04
18:0_22:4 PE	0.24	0.008
18:0_22:5 PE	0.33	0.0014
18:0_22:6 PE	0.33	0.0011
18:0_18:1 PI	0.62	<0.0001
18:0_18:2 PI	0.75	<0.0001
18:0_20:4 PI	0.22	0.01
18:0_22:4 PI	0.34	0.001
18:0_22:5 PI	0.22	0.01
18:0_22:6 PI	0.56	<0.0001

## Data Availability

All data for this study are provided in the paper; no archived datasets were used.

## Supplementary Materials

The following supporting information can be downloaded at: https://www.mdpi.com/article/10.3390/nu16172934/s1, Table S1: Placental Phosphatidylethanolamine Abundance (pmol/ug protein); Table S2: Placental Phosphatidylcholine Abundance (pmol/ug protein); Table S3: Placental Phosphatidylinositol Abundance (pmol/ug protein); Table S4: Placental Phosphatidic Acid Abundance (pmol/ug protein).
