# Supplementary material for: Maternal Docosahexaenoic Acid Supplementation Alters Maternal and Fetal Docosahexaenoic Acid Status and Placenta Phospholipids in Pregnancies Complicated by High Body Mass Index
Source: Nutrients. 2024 Sep 2;16(17):2934. doi: 10.3390/nu16172934 (PMC11397315; doi:10.3390/nu16172934)
Supplement: Supplementary file 1 [file nutrients-16-02934-s001.zip › nutrients-3156442-supplementary.pdf]

Supplemental Table S1	Placental Phosphatidylethanolamine Abundance (pmol/ug protein)										
	Control				Obese				P Values		
	0		200		0		200				
Lipid	Average	SEM	Average	SEM	Average	SEM	Average	SEM	Interaction	Supplementation	BMI
16:0_16:0PE	0.729	0.08	0.680	0.08	1.051	0.08	0.650	0.12	0.0594	0.018	0.1129
16:0_16:1PE	0.121	0.02	0.126	0.02	0.154	0.02	0.080	0.01	0.0419	0.0754	0.7445
16:0_18:0PE	0.078	0.01	0.071	0.00	0.127	0.01	0.069	0.01	0.0042	0.0005	0.007
16:0_18:1PE	1.498	0.14	1.662	0.21	2.393	0.27	1.349	0.17	0.0048	0.0333	0.1492
16:0_18:2PE	2.167	0.23	2.106	0.38	3.429	0.55	1.859	0.28	0.0455	0.0318	0.1695
16:0_20:4PE	5.155	0.35	6.296	0.77	8.168	0.79	5.001	0.83	0.0032	0.1368	0.2047
16:0_22:4PE	0.264	0.04	0.264	0.02	0.320	0.03	0.235	0.03	0.2231	0.2139	0.685
16:0_22:5PE	0.317	0.04	0.294	0.05	0.395	0.05	0.239	0.03	0.1455	0.0558	0.7964
16:0_22:6PE	0.705	0.11	0.823	0.12	0.958	0.11	0.810	0.20	0.3486	0.9161	0.3986
18:0_18:0PE	1.059	0.13	1.258	0.15	1.549	0.16	1.121	0.20	0.0618	0.4804	0.2802
18:0_18:1PE	1.278	0.13	1.394	0.15	1.925	0.26	1.145	0.15	0.0139	0.0609	0.2516
18:0_18:2PE	2.917	0.37	3.015	0.53	5.034	1.03	2.682	0.35	0.0461	0.0651	0.1394
18:0_20:4PE	11.410	0.89	13.039	1.22	17.547	2.11	10.603	1.26	0.0039	0.0596	0.1815
18:0_20:5PE	0.024	0.00	0.032	0.01	0.038	0.01	0.022	0.01	0.1139	0.5804	0.7542
18:0_22:4PE	0.488	0.07	0.508	0.05	0.607	0.08	0.442	0.05	0.1913	0.3012	0.7023
18:0_22:5PE	0.696	0.09	0.625	0.09	0.862	0.12	0.572	0.07	0.2644	0.0724	0.5635
18:0_22:6PE	1.118	0.20	1.401	0.22	1.754	0.29	1.461	0.35	0.2838	0.9857	0.1981
18:1_18:1PE	1.394	0.16	1.748	0.34	2.083	0.29	1.016	0.11	**0.0063**	0.1473	0.9283
18:1_18:2PE	1.693	0.19	1.794	0.41	2.841	0.49	1.394	0.19	**0.025**	**0.0484**	0.2604
18:1_20:4PE	3.212	0.33	3.882	0.48	4.595	0.48	2.813	0.38	**0.0073**	0.1971	0.7113
18:1_22:4PE	0.231	0.03	0.237	0.03	0.300	0.03	0.177	0.02	**0.0464**	0.07	0.9019
18:1_22:5PE	0.526	0.08	0.556	0.13	0.701	0.11	0.385	0.05	0.0863	0.153	0.9877
18:1_22:6PE	1.254	0.27	1.700	0.31	1.795	0.14	1.478	0.40	0.2204	0.8334	0.6024

Supplemental Table S2 Placental Phosphatidylcholine Abundance (pmol/ug protein)											
	**Control**				**Obese**				**P Values**		
	**0**		**200**		**0**		**200**				
**Lipid**	**Average**	**SEM**	**Average**	**SEM**	**Average**	**SEM**	**Average**	**SEM**	**Interaction**	**Supplementation**	**BMI**
16:0_16:0PC	33.242	2.69	39.159	3.57	51.060	6.64	32.965	5.00	**0.0302**	0.1795	0.1026
16:0_16:1PC	1.832	0.17	2.152	0.29	2.679	0.27	1.599	0.23	**0.0218**	0.121	0.3602
16:0_18:0PC	0.728	0.06	0.811	0.07	1.112	0.17	0.728	0.09	**0.0499**	0.1422	0.0802
16:0_18:1PC	8.338	0.85	9.936	1.04	13.156	1.53	8.335	0.89	**0.0193**	0.1543	0.0806
16:0_18:2PC	17.253	1.32	18.240	2.38	29.528	5.94	16.409	2.16	**0.0459**	0.0599	0.0713
16:0_20:4PC	44.121	3.23	52.134	4.61	74.369	7.04	41.439	4.78	**0.0008**	**0.0136**	**0.0297**
16:0_20:5PC	0.204	0.03	0.241	0.04	0.307	0.08	0.198	0.04	0.1862	0.4408	0.4143
16:0_22:4PC	0.570	0.09	0.591	0.05	0.666	0.10	0.550	0.08	0.6799	0.6264	0.4977
16:0_22:5PC	0.607	0.07	0.541	0.08	0.719	0.07	0.463	0.06	0.3936	0.0411	0.5294
16:0_22:6PC	1.320	0.20	1.603	0.20	1.973	0.33	1.489	0.29	0.2683	0.7369	0.1735
18:0_18:0PC	0.063	0.01	0.074	0.01	0.074	0.01	0.076	0.02	0.7995	0.5533	0.3377
18:0_18:1PC	1.106	0.11	1.263	0.09	1.590	0.26	1.068	0.14	0.0942	0.2571	0.1957
18:0_18:2PC	4.197	0.42	4.344	0.59	7.112	1.72	4.007	0.59	0.1114	0.1074	0.1066
18:0_20:4PC	17.395	1.39	19.583	1.73	25.474	3.40	15.599	1.70	**0.0192**	0.0724	0.2053
18:0_20:5PC	0.067	0.01	0.079	0.01	0.097	0.02	0.062	0.01	0.1803	0.4346	0.559
18:0_22:4PC	0.246	0.04	0.253	0.03	0.250	0.05	0.214	0.04	0.9107	0.7355	0.9944
18:0_22:5PC	0.246	0.03	0.219	0.03	0.238	0.02	0.178	0.02	0.9772	0.1911	0.7819
18:0_22:6PC	0.432	0.07	0.516	0.07	0.614	0.12	0.464	0.09	0.31	0.7256	0.3101
18:1_18:1PC	1.288	0.13	1.714	0.21	2.070	0.33	1.118	0.13	**0.0079**	0.183	0.4452
18:1_18:2PC	3.250	0.29	3.652	0.48	5.898	1.31	3.119	0.43	**0.0374**	0.0834	0.0876
18:1_20:4PC	10.187	0.87	13.050	0.77	15.846	1.56	9.538	1.11	**0.0011**	0.1071	0.2121
18:1_20:5PC	0.029	0.01	0.032	0.01	0.041	0.01	0.030	0.01	0.5167	0.6449	0.4355
18:1_22:4PC	0.114	0.02	0.122	0.01	0.134	0.03	0.111	0.02	0.5834	0.7007	0.6151
18:1_22:5PC	0.167	0.02	0.154	0.03	0.188	0.03	0.123	0.02	0.5373	0.1519	0.8747
18:1_22:6PC	0.325	0.07	0.420	0.07	0.477	0.06	0.396	0.08	0.3745	0.8842	0.2467

Supplemental Table S3 Placental Phosphatidylinositol Abundance (pmol/ug protein)											
	Control				Obese				P Values		
	0		200		0		200				
Lipid	Average	SEM	Average	SEM	Average	SEM	Average	SEM	Interaction	Supplementation	BMI
16:0_16:0PI	0.399	0.05	0.334	0.15	0.762	0.19	0.193	0.04	**0.0334**	**0.0064**	0.2885
16:0_16:1PI	0.042	0.01	0.042	0.02	0.081	0.01	0.008	0.00	**0.0045**	**0.0016**	0.6721
16:0_18:0PI	0.117	0.02	0.098	0.04	0.241	0.08	0.061	0.01	**0.0558**	**0.0179**	0.2711
16:0_18:1PI	0.918	0.10	0.935	0.28	2.096	0.41	0.400	0.05	**0.0008**	**0.0006**	0.1304
16:0_18:2PI	0.474	0.04	0.356	0.10	1.107	0.27	0.234	0.03	**0.0059**	**0.0005**	0.0509
16:0_20:4PI	7.584	0.76	7.291	1.73	16.239	2.37	3.625	0.26	**<0.0001**	**<0.0001**	0.0835
16:0_22:4PI	0.313	0.04	0.296	0.09	0.620	0.09	0.114	0.01	**0.0007**	**0.0003**	0.3121
16:0_22:5PI	0.317	0.05	0.230	0.07	0.596	0.09	0.095	0.01	**0.0019**	**<0.0001**	0.2415
16:0_22:6PI	0.235	0.03	0.199	0.04	0.598	0.14	0.128	0.02	**0.0029**	**0.0018**	0.0632
18:0_18:0PI	0.021	0.00	0.025	0.01	0.046	0.01	0.008	0.00	**0.0057**	**0.016**	0.5175
18:0_18:1PI	0.190	0.02	0.244	0.09	0.408	0.09	0.096	0.01	**0.0029**	**0.0154**	0.4257
18:0_18:2PI	0.358	0.03	0.320	0.10	0.820	0.22	0.210	0.03	**0.0119**	**0.0042**	0.0966
18:0_20:4PI	16.127	1.15	15.756	4.16	30.816	6.26	8.516	0.64	**0.0027**	**0.0016**	0.2422
18:0_22:4PI	0.283	0.02	0.294	0.10	0.504	0.10	0.128	0.02	**0.0049**	**0.0068**	0.6519
18:0_22:5PI	0.211	0.02	0.162	0.05	0.348	0.06	0.075	0.01	**0.0054**	**0.0001**	0.4763
18:0_22:6PI	0.188	0.02	0.159	0.03	0.442	0.10	0.122	0.02	**0.0049**	**0.0027**	0.057
18:1_18:1PI	0.181	0.02	0.259	0.10	0.353	0.06	0.074	0.01	**0.0021**	**0.0373**	0.9568
18:1_18:2PI	0.112	0.02	0.104	0.04	0.224	0.05	0.047	0.01	**0.0083**	**0.004**	0.3484
18:1_20:4PI	1.496	0.14	1.448	0.38	2.648	0.36	0.662	0.09	**0.0006**	**0.0003**	0.417
18:1_22:4PI	0.014	0.00	0.029	0.02	0.017	0.00	0.004	0.00	0.1129	0.9804	0.2091

Supplemental Table S4 Placental Phosphatidic Acid Abundance (pmol/ug protein)											
	Control				Obese						
	0		200		0		200		P Values		
**Lipid**	**Average**	**SEM**	**Average**	**SEM**	**Average**	**SEM**	**Average**	**SEM**	**Interaction**	**Supplementation**	**BMI**
16:0_16:0PA	0.0028	0.0004	0.0056	0.0026	0.0034	0.0004	0.0038	0.0022	0.48	0.31	0.66
16:0_18:1PA	0.0009	0.0001	0.0023	0.0010	0.0024	0.0005	0.0017	0.0013	0.2	0.79	0.48
16:0_18:2PA	0.0006	0.0001	0.0016	0.0007	0.0017	0.0003	0.0013	0.0010	0.19	0.6	0.5
16:0_20:4PA	0.0089	0.0008	0.0226	0.0079	0.0171	0.0014	0.0161	0.0082	0.16	0.23	0.87
16:0_22:6PA	0.0007	0.0002	0.0021	0.0007	0.0020	0.0005	0.0017	0.0008	0.18	0.41	0.41
18:0_18:1PA	0.0031	0.0004	0.0049	0.0011	0.0120	0.0020	0.0040	0.0009	**0.0001**	**0.0091**	**0.0006**
18:0_18:2PA	0.0024	0.0003	0.0035	0.0008	0.0092	0.0017	0.0027	0.0008	**0.0004**	**0.0119**	**0.0047**
18:0_20:4PA	0.0213	0.0013	0.0378	0.0085	0.0588	0.0086	0.0360	0.0129	**0.018**	0.69	**0.03**
18:0_22:4PA	0.0026	0.0004	0.0033	0.0006	0.0065	0.0011	0.0024	0.0007	**0.0011**	**0.015**	**0.048**
18:0_22:5PA	0.0026	0.0004	0.0034	0.0007	0.0078	0.0016	0.0031	0.0004	**0.0017**	**0.0149**	**0.0063**
18:0_22:6PA	0.0046	0.0007	0.0082	0.0011	0.0153	0.0031	0.0071	0.0014	**0.0014**	0.1498	**0.0049**
18:1_20:4PA	0.0016	0.0003	0.0042	0.0019	0.0032	0.0007	0.0031	0.0014	0.3414	0.3414	0.816
